# Efficacy and Safety of Artemether-Lumefantrine in the Treatment of Acute, Uncomplicated *Plasmodium falciparum* Malaria: A Pooled Analysis

**DOI:** 10.4269/ajtmh.2011.11-0069

**Published:** 2011-11-01

**Authors:** Michael Makanga, Quique Bassat, Catherine O. Falade, Zulfiqarali G. Premji, Srivicha Krudsood, Philip Hunt, Verena Walter, Hans-Peter Beck, Anne-Claire Marrast, Marc Cousin, Philip J. Rosenthal

**Affiliations:** European and Developing Countries Clinical Trials Partnership, Cape Town, South Africa; Barcelona Center for International Health Research, Hospital Clínic/Institut d'Investigacions Biomèdiques August Pi i Sunyer, University of Barcelona, Barcelona, Spain; Centro de Investigação em Saúde de Manhiça, Manhiça, Maputo, Mozambique; Department of Pharmacology and Therapeutics, University of Ibadan, Ibadan, Nigeria; Muhimbili University of Health and Allied Sciences, Dar-es-Salaam, Tanzania; World Health Organization Collaborating Centre for Clinical Management of Malaria, Faculty of Tropical Medicine, Mahidol University, Bangkok, Thailand; Novartis Pharma, Horsham, United Kingdom; Novartis Pharma AG, Basel, Switzerland; Swiss Tropical and Public Health Institute and University of Basel, Basel, Switzerland; Department of Medicine, University of California, San Francisco, California

## Abstract

Randomized trials have confirmed the efficacy and safety of artemether-lumefantrine (AL) for treatment of uncomplicated *Plasmodium falciparum* malaria. Data from seven studies supported by Novartis (1996–2007), including 647 adults (> 16 years of age, 83.3% completed the study) and 1,332 children (≤ 16 years of age, 89.3% completed the study) with microscopically confirmed uncomplicated *P. falciparum* malaria and treated with the recommended regimen of AL, were pooled. The 28-day polymerase chain reaction–corrected parasitologic cure rate (primary efficacy endpoint) was 97.1% (495 of 510) in adults and 97.3% (792 of 814) in children (evaluable population). Gametocytemia prevalence after day was 4.2% (23 of 554) in adults and 0.9% (8 of 846) in children. No noteworthy safety signals were observed. Serious adverse events occurred in 1.4% of the adults and 1.3% of the children. This study is the largest data set to date assessing AL therapy for treatment of acute uncomplicated *P. falciparum* malaria. Artemether-lumefantrine showed high cure rates and rapid resolution of parasitemia, fever, and gametocytemia in adults and children, and showed an excellent safety and tolerability profile.

## Introduction

The World Health Organization (WHO) recommends that artemisinin-based combination therapies (ACTs) be used as first-line treatment of uncomplicated *Plasmodium falciparum* malaria.^1^ Treatment with ACTs is currently considered more effective than available non-artemisinin regimens,^2^ resulting in faster symptomatic improvement and parasite clearance^1^,^3^ and a reduction in gametocyte carriage, which could help to reduce malaria transmission.^1^,^4^–^6^

In 2004, artemether-lumefantrine (AL, Coartem^®^, Novartis Pharma AG, Basel, Switzerland) became the first ACT to be pre-qualified by WHO, and it is now used as first-line treatment for uncomplicated *Plasmodium falciparum* malaria in many regions worldwide.^7^ The combination of artemether, which rapidly reduces parasite biomass,^8^ with longer-acting lumefantrine, capable of eliminating residual parasites,^9^ has proven to be highly effective in achieving parasitologic cure, symptom relief,^10^–^15^ and reduction of gametocyte carriage.^15^,^16^ Typically, although with uncommon exceptions,^17^ the 28-day parasitologic cure observed after AL therapy exceeded 95% in evaluable patients,^10^–^15^,^18^–^20^ meeting the WHO recommendation that cure rates for *P. falciparum* malaria should be at least 90% and preferably > 95%.^1^

Although numerous randomized, controlled trials have confirmed the efficacy and safety of AL for treatment of uncomplicated *P. falciparum* malaria in regions worldwide,^10^–^14^,^20^–^28^ further analyses are desirable to ascertain whether efficacy is affected by different patient characteristics and to identify any infrequent safety concerns not detected within the limited sizes of individual trials. It is useful to examine data separately for adults and children because of differences between these groups in antimalarial immunity,^29^,^30^ transmission intensity, and disposition of some antimalarial drugs.^31^

A recent Cochrane analysis evaluated the efficacy of ACTs for the treatment of uncomplicated *P. falciparum* malaria and confirmed that the polymerase chain reaction (PCR)–corrected parasitologic cure rate for AL at day 28 was > 95% in all the trials reviewed.^2^ However, this analysis did not assess efficacy or safety outcomes separately among adults, children, or other subpopulations. Two pooled analyses of outcomes with AL have been performed in which children and adults were studied and the populations were defined by age,^18^,^32^ but the cut-off point used (12 years) is not the one recommended by the U.S. Food and Drug Administration (FDA) (i.e., 16 years).^6^

We report efficacy and safety findings for AL in a post-hoc pooled analysis of data from the Novartis study database, which includes approximately 2,000 children and adults treated for uncomplicated *P. falciparum* malaria during 1996–2007. The analysis population was derived from six studies undertaken in Asia and Africa and one study in patients from Europe and Colombia. This, it encompasses regions with varying endemicity and transmission patterns.

## Materials and Methods

### Study identification and design.

Data from clinical trials within the Novartis database were included in this unplanned pooled analysis if they enrolled patients treated with the recommended regimen of AL, administered twice a day for three days, and diagnosis of *P. falciparum* malaria was based on microscopically confirmed evaluation of Giemsa-stained blood slides. Patients with severe or complicated malaria were not included in this analysis. Study B2303 used an investigator-blinded design and A025 used a double-blind design; the remaining studies were open label. Six of the studies were performed in malaria-endemic countries (studies A025, A026, A028, and A2412 in Thailand; A2403 and B2303 in Africa)^19^,^26^,^27^,^33^–^35^ and one involved non-immune adult travelers in Europe and non-endemic regions of Colombia (A2401).^36^ The B2303 study was a randomized trial of the AL dispersible formulation versus AL tablets administered crushed: patients receiving the dispersible tablet were only included in the safety analyses (data from the B2303 study population confirmed equivalent efficacy and safety of the dispersible formulation and the crushed AL tablets).^34^ The A2401 study included only adults. Studies A2403 and B2303 included only children (< 10 years of age and ≤ 12 years of age, respectively), and were both conducted in Africa (A2403: Kenya, Nigeria, and Tanzania; B2303: Benin, Kenya, Mali, Mozambique, and Tanzania). Three of the trials (A025, A026, and A028) took places during 1996–1999, and four took place during 2001–2007 (A2401, A2403, B2303, and A2412).

In all studies, AL dosing was based on body weight: 5–< 15 kg, 1 tablet per dose; 15–< 25 kg, 2 tablets per dose; 25–< 35 kg, 3 tablets per dose; ≥ 35 kg, 4 tablets per dose. Patients who vomited the first dose within one hour of treatment received a full replacement in all studies. The protocols of A2403 and B2303 specified that no more than two doses were to be replaced during the entire treatment period.

Six of the studies (A025, A026, A028, A2403, B2303, and A2401) recorded the 28-day parasitologic cure rate, corrected for re-infection by PCR in studies A025, A026, A028, A2403, and B2303 (as defined below under Data analysis), consistent with draft guidance from the FDA. The PCR analysis was performed by analysis of size polymorphisms in the *P. falciparum* genes encoding merozoite surface protein 2, merozoite surface protein 1, and glutamate-rich protein^37^,^38^ in studies A025, A026, A028, A2403, and B2303. The PCR analysis was not performed in study A2401 because the patients were travelers from regions to which malaria was not endemic and who were not at risk of a new infection. In this study, any recurring parasites were regarded as recrudescences. These studies also recorded time to parasite and fever clearance, as defined below (see Data analysis). The remaining study (A2412) was an open-label, single-center, safety study and was included only in the safety analysis. Patients were followed-up for at least 28 days in all studies. Giemsa-stained thick blood smears were examined for asexual forms and gametocytes on days 0–7 (day 0, first day of AL treatment) and thereafter on days 14, 21, 28, and 42. All doses of AL were given under supervision in all studies except A2401.

### Data analysis.

The main efficacy variable was the 28-day PCR-corrected parasitologic cure rate, which was defined as the proportion of patients with clearance of asexual parasitemia within 7 days of the first dose of AL and without subsequent recrudescence within 28 days. Recrudescence was defined as reappearance of the original parasite strain as confirmed by PCR-based genotyping. Other efficacy endpoints were the 28-day non–PCR-corrected parasitologic cure rate, time to parasite clearance, time to fever clearance, and proportion of patients with gametocytes at specific time points. The times at which patients were assessed for parasite clearance and fever clearance varied between studies. The presence of gametocytes was recorded at baseline, > 0–72 hours, > 72 hours–day 7, and > day 7.

Safety data included the incidence, type, and study drug relationship of adverse events. Adverse events were defined as the appearance or worsening of any undesirable sign, symptom, or medical condition after starting the study drug, even if the event was not considered to be related to the study drug, according to the International Conference on Harmonization Guidelines. A serious adverse event was defined as any untoward medical occurrence that resulted in death, was life-threatening, required inpatient hospitalization or prolongation of existing hospitalization, resulted in persistent or significant disability/incapacity, or was a congenital anomaly/birth defect. Other safety data included the incidence of death and serious adverse events, clinical laboratory assessments, and vital signs. The availability of laboratory data varied between studies in terms of parameters measured and time of sample collection. Creatinine clearance was used as a measure of renal function, and was calculated according to the Cockcroft-Gault formula in adults^39^ and the Shull formula in children.^40^

### Statistical analysis.

Patients > 16 years of age were included in the adult analysis populations; patients ≤ 16 years of age were included in the pediatric analysis population. Efficacy and safety data were taken from the original study databases.

Efficacy analyses were based on the modified intent-to-treat (mITT) population, which was generally defined as all patients with parasitologically confirmed *P. falciparum* malaria who received at least one dose of study drug. This definition was selected to comply with FDA draft guidance.^41^ Efficacy analyses were also performed for evaluable patient populations. Definitions of the evaluable patient population differed between studies, but in general the evaluable population included patients from the mITT population who took no other drug that had an anti-malarial effect, and had parasite counts recorded up to day 28, or who discontinued the study because of an unsatisfactory therapeutic effect. A *post hoc* analysis of the primary efficacy endpoint, 28-day PCR-corrected parasitologic cure rate, was performed on the basis of data only from study A2401 by using the last observation carried forward technique.

The 28-day PCR-corrected parasitologic cure rate was also reported for subpopulations of the evaluable population, according to the following variables: 1) baseline parasite count (≤ 100,000/μL or > 100,000/μL); 2) sex; 3) baseline renal function based on creatinine clearance (normal > 80 mL/min; mild dysfunction ≥ 50–≤ 80 mL/min; moderate dysfunction ≥ 30–< 50 mL/min; or severe dysfunction < 30 mL/min) (creatinine clearance < 30 mL/min is indicative of severe malaria in adults and no adult patients with severe renal dysfunction were recruited, in adherence with the studies' exclusion criteria); 4) baseline hepatic function (normal, total bilirubin ≤ upper limit of normal [ULN], and aspartate aminotransferase [AST] ≤ ULN if available; mild dysfunction, total bilirubin ≤ ULN and AST > ULN, or total bilirubin > ULN – 1.5 × ULN; moderate dysfunction, total bilirubin > 1.5 – 3 × ULN; severe dysfunction, total bilirubin > 3 × ULN; if the AST value was missing, baseline alanine aminotransferase value was used as per the criteria for AST); and 5) age (pediatric population only) (> 1 month–≤ 2 years, > 2–≤ 16 years, or >12–≤ 16 years) and body weight (5–< 10 kg, 10–< 15 kg, 15–< 25 kg, 25–< 35 kg, or ≥ 35 kg).

All data are presented descriptively. One-sample 95% confidence intervals (CIs) for the 28-day PCR-corrected cure rate were calculated by using the Pearson-Clopper method. Kaplan-Meier estimates of parasite clearance time and fever clearance time were calculated. Laboratory data are presented as standard summary statistics. Because of the variation between studies in the time points used for laboratory evaluations and deviations from schedules, time windows were defined. If a patient had more than one value within a given interval, the mean was used for summarization. Summary statistics for laboratory data should be interpreted carefully in view of the variation between time windows and the numbers of patients with available data. Safety analyses were based on all randomized/enrolled patients who received at least one dose of study drug. Statistical analyses were performed using the SAS software system (SAS Institute, Cary, NC).

### Study conduct.

All studies were conducted according to the Declaration of Helsinki, and informed consent was obtained from all patients (or parents/guardians where appropriate) according to appropriate institutional review board approval.

## Results

### Populations for analysis and patient disposition.

**Figure 1.**
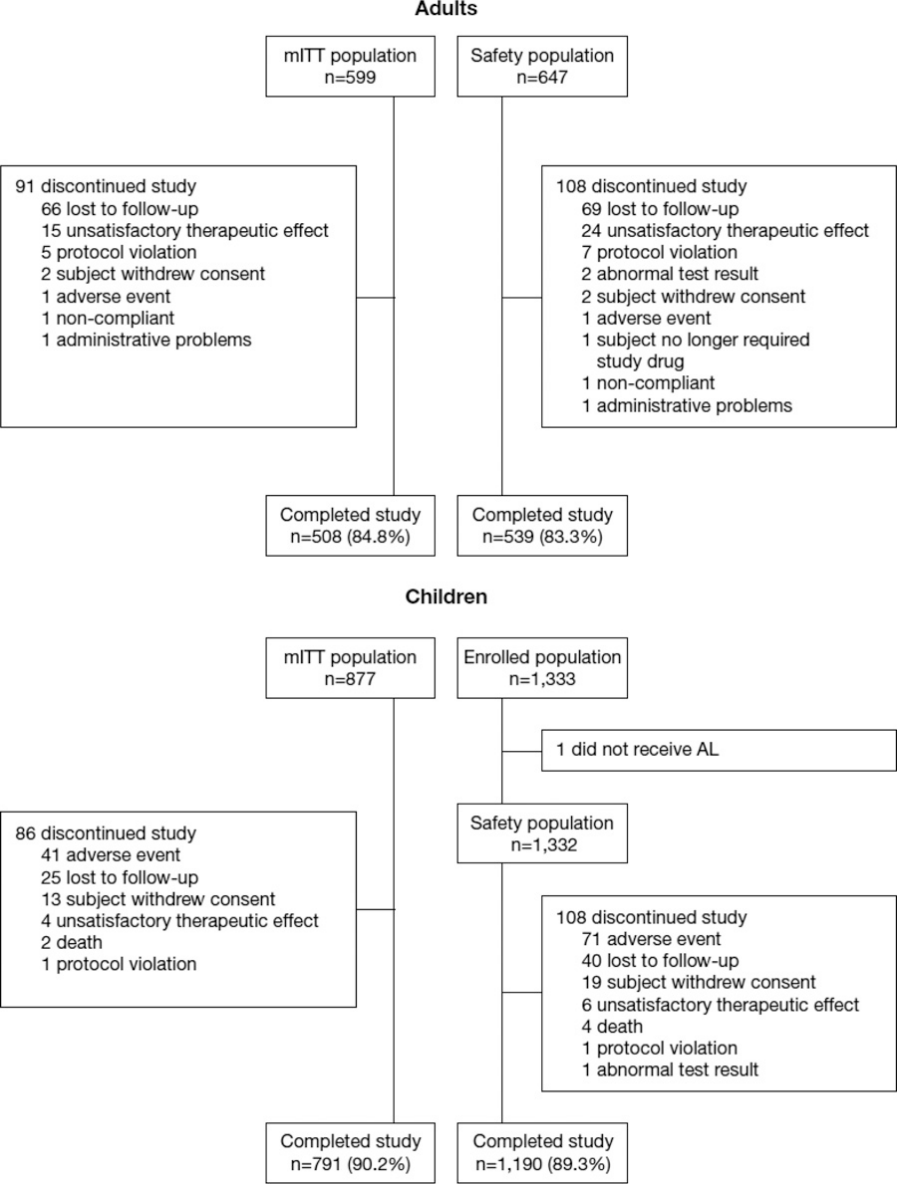
Patient disposition for adults and children in the study of artemether-lumefantrine for treatment of uncomplicated *Plasmodium falciparum* malaria.

For adults, the mITT population comprised 599 patients, of whom 437 took part in studies from malaria-endemic regions (studies A025, A026, and A028), and 162 were travelers from non-endemic regions (study A2401) (Table 1). The evaluable population comprised 513 patients. Patients who received at least one dose of AL were considered for safety evaluation. The safety population comprised 647 patients, including 44 from study A2412, who were not included in the efficacy analysis because efficacy was addressed only as secondary objective and the study was stopped early for administrative reasons. For children (age ≤ 16 years), the mITT population comprised 877 patients and the evaluable population comprised 828 patients. A total of 1,333 pediatric patients were enrolled, but one patient in study A2412 did not receive AL and was excluded from the safety population. In study B2303, 447 patients received dispersible AL and were included in the safety, but not in the efficacy analysis. For children and adults, at least 83% of the mITT and safety populations completed the study (Figure 1).

Patient demographics and baseline disease characteristics are shown in Table 2. Information on race was not collected in studies A025, A026, or A028, which studied populations in Thailand. In the pediatric population, more than 90% of patients were ≤ 12 years of age; almost half were 1 month–2 years of age. A total of 29 children in the pediatric mITT population (3.3%), 28 patients in the pediatric evaluable population (3.4%), and 36 patients in the pediatric safety population (2.7%) were 1–6 months of age. Approximately 90% of adults and children for whom parasite counts were available had a count ≤ 100,000/μL.

### Exposure to AL.

The full recommended regimen of AL was taken by 96.9% of adults (n = 627) and 97.1% of children (n = 1,332) (safety populations). Seven adults (1.1%) and 140 children (10.5%) vomited at least one dose of AL.

### Parasitologic cure.

Parasitologic cure rates on day 28 among the pooled efficacy population are summarized in Table 3. In adults, the 28-day PCR-corrected parasitologic cure rate was 97.1% (495 of 510; 95% CI = 95.2–98.3%) in the evaluable population and 83.4% (499 of 598; 95% CI = 80.2–86.3%) in the mITT population. The difference in results was caused primarily by study A2401, the only study of non-immune travelers, in which a large number of patients did not undergo parasitologic assessments as scheduled. In this study, 33 of the 42 patients showed treatment failure. These results were caused by missing parasite assessments at day 28; negative blood smears were available at earlier time points for 27of these persons. An additional five treatment failures were so-classified because the patients received other antimalarial medications. Thus, in study A2401, the mITT cure rate was 74.1% (120 of 162; 95% CI = 66.6–80.6%), and the evaluable cure rate was 96.0% (119 of 124; 95% CI = 90.8–98.7%); a *post hoc* analysis using the last observation carried forward technique showed a 90.7% cure rate (147 of 162).

One potential explanation for treatment failures may be underdosing of larger patients. In the evaluable population of study A2401, the cure rate was 93.0% (66 of 71; 95% CI = 84.3–97.7%) in patients weighing ≥ 70 kg versus 100% (52 of 52; 95% CI = 93.2–100%) in patients < 70 kg. Of the five patients weighing ≥ 70 kg who were classified as treatment failures, one patient discontinued treatment after the second dose because of signs and symptoms of severe malaria that were already present at baseline, one missed an assessment at day 6 but was clear of parasites at day 7, and three had recrudescence during days 21 and 28. In addition, of four patients with body weights >100 kg, three were clear of parasites at day 28, and one did not have a day 28 blood assessment, but was parasite free at 58 and 154 hours.

In the pediatric population, the 28-day PCR-corrected parasitologic cure rate was 97.3% (792 of 814; 95% CI = 95.9–98.3%) in the evaluable population and 93.4% (798 of 854; 95% CI = 91.6–95.0%) in the mITT population. No differences were observed between age groups or different body weight categories, including patients ≤ 2 years of age or those weighing < 10 kg (Table 4).

Baseline parasite density did not appear to affect the 28-day PCR-corrected cure rate in either adults or children (Table 4). There was a trend towards a lower cure rate in patients with impaired hepatic function in the adult and pediatric populations, but there were few patients with moderate/severe impairment (only 13 adults and 2 children with severe impairment), which limited the reliability of these findings (Table 4).

### Parasite clearance time.

The median time to parasite clearance was 42.3 hours (95% CI = 41.5–43.2 hours) in the adult mITT population, and parasite clearance was achieved within 48 hours in 78.6% of patients (Table 5). For children, the median parasite clearance time for the mITT population was 35.3 hours (95% CI = 31.7–35.7 hours), and 90.3% of patients showed parasite clearance in ≤ 48 hours. In the study A2401 mITT analysis, the median time to parasite clearance was 41.8 hours (95% CI = 40.3–43.8 hours), and 108 (066.7%) of 162 patients showed parasite clearance ≤ 48 hours.

### Fever clearance time.

In adults, the median time to fever clearance was 28.5 hours (95% CI = 22.3–34.0 hours). Median time to fever clearance was shorter in patients with a baseline parasite count ≤ 100,000/μL (n = 464, 22.1 hours; 95% CI = 21.2–24.0 hours) compared with those with a baseline count > 100,000/μL (n = 34, 43.1 hours; 95% CI = 21.7–45.9 hours). The median fever clearance time in children (7.9 hours; 95% CI = 7.9–8.0 hours) was markedly shorter than that in adults (28.5 hours; 95% CI = 22.3–34.0 hours). As in adults, a low baseline *P. falciparum* count (≤ 100,000/μL) was associated with slightly shorter fever clearance time (n = 788, 7.9 hours; 95% CI = 7.8–8.0 hours) compared with those with parasite counts > 100,000/μL (n = 88, median 11.3 hours; 95% CI = 7.9–23.4 hours). For study A2401, the median time to fever clearance among patients with fever at baseline was 36.5 hours (95% CI = 27.8–39.5 hours in the mITT population).

### Gametocyte carriage.

The proportion of adult patients with *P. falciparum* gametocytes at baseline was 9.7% (58 of 596), which decreased to 4.2% (23 of 554) after day 7 (Table 5). Among children, 45 (5.1%) of 877 patients had gametocytes at baseline and 8 (0.9%) of 846 patients had gametocytes after day 7. For study A2401, 11.9% (19 of 160) and 1.4% (2 of 139) of patients had gametocytes at baseline and after day 7, respectively. Primaquine, which has gametocytocidal activity and could be prescribed at the discretion of the investigator,^42^ was administered to less than 1% of the patients, mostly after day 7 to treat concomitant *P. vivax* infections or to eliminate *P. falciparum* gametocytes.

### Adverse events in adults.

One or more adverse events were reported by 557 (86.1%) of 647 adult patients. The most frequently reported adverse events were non-specific (headache, anorexia, dizziness, asthenia, arthralgia, myalgia, and nausea) and consistent with symptoms of acute malaria (Table 6). Adverse events potentially related to hypersensitivity reactions were reported by 4.0% of adults (rash = 3.2%, urticaria = 0.6%, facial edema = 0.2%). These adults showed an incidence of 2.8% during days 0–3 of treatment and 1.0% during days 4–10 of treatment (Table 7). Nervous system disorders, mainly headache (55.6%) and dizziness (39.1%), occurred in 59.7% of adults. These results are also consistent with malaria symptoms. Ear and labyrinth disorders were reported for 29 patients (4.5%), of whom 21 experienced vertigo. Prolonged QT interval on electrocardiogram was reported for two adult patients (0.3%); however, syncope, sudden cardiac death, seizure, or significant ventricular arrhythmias was not reported for these patients. One adult patient (1 of 647, 0.2%) stopped treatment prematurely because of adverse events (mild abdominal pain/diarrhea).

No adult patient died. Nine patients (1.4%) experienced 22 serious adverse events. No type of serious adverse event occurred in more than one patient except for *P. falciparum* infection, which was reported by investigators as a serious adverse event in two patients. Of the 22 serious adverse events, 18 occurred in study A2401, in which prolongation of hospitalization required a classification of serious adverse event. There were no cases of urticaria or anaphylactoid reaction reported as serious adverse events. Seven serious adverse events in three patients had a suspected relation to AL (abnormal liver function test results in one patient; disease progression/increased bilirubin/increased transaminases/mental impairment/vomiting in one patient; and recrudescence in one patient). An additional 13 serious adverse events were not suspected to be related to AL, and the relationship with AL was unknown for the final two events.

The overall incidence of adverse events in patients with normal renal function or mild impairment was 77.8% (175 of 225) and 78.3% (112 of 143), respectively. The small number of patients with moderate renal impairment (n = 10) each reported one or more adverse events. Subgroup analysis by hepatic function at baseline showed that the incidence of adverse events in adult patients with normal hepatic function or mild, moderate, or severe hepatic impairment at baseline was 71.9% (120 of 167), 79.3% (96 of 121), 83.3% (50 of 60), and 100% (17 of 17), respectively. Many of the adverse events that occurred more frequently in the presence of hepatic impairment were well-recognized symptoms of malaria.

### Adverse events in children.

One or more adverse events were reported in 970 (72.8%) of 1,332 children; pyrexia (28.6%) and cough (22.7%) occurred most frequently (Table 6). Adverse events potentially related to hypersensitivity reactions occurred in 4.2% of children; the most frequent reactions were rash (2.9%) and dermatitis (0.4%), which showed an incidence of 1.1% during days 0–3 of treatment and 1.0% during days 4–10 of treatment (Table 7). Nervous system disorders were reported as adverse events in 193 patients (14.5%), of which headache (n = 168, 12.6%), dizziness (n = 56, 4.2%), and clonus (n = 11, 0.8%) occurred in > 0.5% of patients. All but one case of clonus were reported at a single center during protocol-specified routine neurologic examinations in which pre-defined signs and symptoms were evaluated, and all cases were graded mild. No reports of clonus occurred in the population of 899 children in the B2303 study. Ear and labyrinth disorders occurred in 6 patients (0.5%), comprising ear pain (n = 3), and cerumen impaction, ear pruritus, and otorrhea in one patient each. Prolonged QT interval by electrocardiogram ECG was reported in one child (0.1%); no other adverse events potentially related to QT prolongation were recorded.

Four (0.3%) children died. Deaths were caused by severe *P. falciparum* infection on day 28 (new infection or recrudescence was undetermined) in a patient who was parasite free ≤ 24 hours and still parasite free on day 13; gastroenteritis on day 8 with diarrhea treated with oral rehydration therapy; an unspecified infection with pyrexia and dehydration treated with quinine, paracetamol, metoclopramide, and amoxicillin; and hemorrhage on day 5 after scarification by a traditional healer; all deaths were considered unrelated to AL by investigators. Thirty serious adverse events were reported by investigators in 17 patients (1.3%), including *P. falciparum* infection (7), convulsion (3), pyrexia (2), and anemia (2). None of the three cases of convulsion was considered to be related to AL; each was reported together with malaria or pyrexia, and may have represented febrile convulsions. All other serious adverse events were reported in no more than one patient. One serious adverse event was reported as having a suspected relationship with AL. This event was a case of urticarial rash in a four-year-old patient that resolved ≤ 5 days after antihistamine treatment.

Seventy-one children (5.3%) left the study prematurely because of adverse events. Seventy of these 71 children were enrolled in study B2303, in which the protocol specified that any patient who required a second replacement dose of study drug was to be removed from the study. Four patients stopped treatment because of an adverse event other than vomiting. These events were *P. falciparum* infection plus anemia in one patient, *P. falciparum* infection plus iron deficiency anemia in another patient, a lower respiratory tract infection in one patient, and urticaria in one patient as described above.

Pediatric patients with mild, moderate, or severe renal impairment at baseline had more adverse events than those without renal impairment (69.4%, 354 of 510; 77.4%, 226 of 292; and 92.2%, 94 of 102 versus 60.7%, 198 of 326). Children with mild or moderate hepatic impairment had a higher incidence of adverse events than those with normal function (77.6%, 132 of 170 and 91.7%, 22 of 24 versus 65.3%, 94 of 144, respectively) but numbers of patients with moderate (n = 24) or severe (n = 2) hepatic impairment were low.

### Laboratory parameters.

Hematogic parameters improved during follow-up, which is consistent with resolution of acute malaria. Mean values ± SD for hemoglobin level, leukocyte count, neutrophil count, and platelet count at baseline were 12.3 ± 2.5 g/dL, 6.1 ± 2.4 × 10^9^/L, 3.9 ± 1.9 × 10^9^/L, and 130 ± 75 × 10^9^/L, respectively, in adults, and 9.3 ± 1.7 g/dL, 9.3 ± 3.8 × 10^9^/L, 4.8 ± 2.8 × 10^9^/L, and 181 ± 99 ×10^9^/L in children. At days 26–39, the corresponding values were 12.8 ± 1.7 g/dL, 7.4 ± 2.2 ×10^9^/L, 3.9 ± 1.7 × 10^9^/L, and 231 ± 81 × 10^9^/L in adults and 10.4 ± 1.4 g/dL, 9.1 ± 3.3 × 10^9^/L, 3.5 ± 1.9 × 10^9^/L, and 310 ± 130 × 10^9^/L in children. Renal function (assessed by creatinine clearance) increased from baseline and liver function values (AST, alanine aminotransferase, and bilirubin) decreased from baseline, which is consistent with malaria and its resolution. Hemolysis-related adverse events were not reported in adults. Four potential hemolysis-related adverse events occurred in the pediatric population (0.3%), all of which were increased reticulocyte counts.

## Discussion

In this pooled analysis, the largest of its type so far to assess AL therapy for the treatment of acute uncomplicated *P. falciparum* malaria, AL achieved high cure rates and rapid symptom relief in adults and children and a good safety and tolerability profile. The efficacy of AL was similar for patients of all ages in malaria-endemic regions. In the only study of travelers from non-endemic regions, analysis was complicated by poor follow-up rates, but efficacy of AL also appeared to be excellent in the per protocol population.^36^ In addition, gametocyte carriage decreased markedly from baseline after AL administration, which suggested that use of AL can contribute to reducing malaria transmission.

The observed day 28 PCR-corrected cure rate in evaluable patients was approximately 97% in adults and children, which exceeded the recommendation of WHO that antimalarial drugs should show a cure rate of 95%.^1^ The cure rate was largely unaffected by age, body weight, baseline parasite count, or kidney function. A trend to lower cure rates in patients with poor baseline liver function may have been an artifact of small patient numbers and/or reflect the development of hepatic impairment in patients with more severe malaria. Efficacy was maintained in the youngest children, who showed a cure rate in evaluable children 1 month–2 years of age of approximately 97%. Importantly, the cure rate was similar in all body weight groups in children, despite the difference in doses received on a milligram per kilogram basis within each dosing category.

Recent reports that showed decreased rates of parasite clearance after treatment with artesunate and ACTs in western Cambodia are of concern.^43^,^44^ However, if one considers this dataset and other recent studies, there is no convincing evidence that the efficacy of AL has changed in recent years.^45^ The seven studies included in our analysis spanned the period 1996–2007, but most adults received AL treatment during 1996–1999, and the largest studies in children took place during 2002–2007. Randomized, multicenter trials during 2005–2009 in Africa^10^,^11^,^14^,^20^,^22^,^23^,^28^,^46^,^47^ and Asia^24^,^25^,^48^ have each demonstrated a PCR-corrected cure rate greater than 95% after AL treatment in a variety of populations and settings. These studies have not included Cambodia, and it is likely that alterations in artemisinin sensitivity that have led to changes in parasite clearance in that country are not yet relevant in most malarious areas. In addition, an advantage of the AL combination is that lumefantrine has never been used as monotherapy for the treatment of malaria,^1^ and resistance to this drug in field isolates has not yet clearly been demonstrated.

Parasite and fever clearance were achieved rapidly after treatment with AL in adult and pediatric populations. The slower fever clearance observed in study A2401 may have been caused by less frequent evaluation in this study and the relatively small proportion of patients (approximately 20%) who used antipyretics compared with other trials (approximately 80% in studies A025, A026, and A028).

The reduction in gametocytemia after treatment with AL is noteworthy. Clearance of gametocytes, the parasite form that infects mosquitoes, breaks the cycle of transmission, and thus contributes to malaria control. Artemisinins have gametocytocidal properties and reduce gametocyte carriage,^4^ and AL has shown significantly greater gametocytocidal activity than antimalarial drugs other than artemisinin.^16^,^27^,^49^–^51^ Randomized trials have demonstrated significantly lower rates of gametocytemia after AL treatment compared with oral quinine^47^ or dihydroartemisinin-piperaquine^50^,^52^ in patients in Africa with uncomplicated *P. falciparum* malaria. In our analysis, the proportion of patients with gametocytemia after day 7 decreased by more than 50% from baseline in adults and by approximately 80% in children. Less than 1% of treated children were positive for gametocytes after day 7.

Our analysis showed no unexpected safety concerns in almost 2,000 patients treated with AL. These results are consistent with those of other studies that demonstrated excellent safety and tolerability of AL.^11^,^28^,^53^,^54^ Most of the frequently reported adverse events were typical signs and symptoms of malaria.^55^ There were few serious adverse events or deaths, and serious adverse events were only rarely suspected to be related to AL therapy. Urticaria was reported as a severe adverse event in only one child and in no adults. Most adverse events related to the nervous system, ear, and labyrinth disorders were transient and reversible, and there were no adverse events associated with QTc prolongation. Hemolysis-related adverse events were absent in adults and rare in the pediatric population (reticulocyte count increase in 0.3% of patients, in parallel with an increase in hemoglobin level) with the few reported events deemed unrelated to AL treatment. Thus, AL treatment does not appear to trigger hemolysis, consistent with the findings reported by Premji and others^56^ in a randomized, double-blind trial of patients in Africa with uncomplicated *P. falciparum* malaria.

Unforeseen differences were not observed in the adverse event profile or laboratory results between subgroups. The higher rate of adverse events reported in patients with hepatic or renal impairment is likely to reflect more severe malaria in these groups. Cough was more frequent in children than in adults, which is not unexpected because respiratory infections are common in children in Africa with malaria.^57^ Previous reports have suggested that cough is unlikely to be causally related to artemesinin-based therapy, and the fact that time to onset of cough was relatively evenly distributed throughout the duration of the studies suggests that cough was probably not related either to malaria or to AL treatment. The higher rates of headache and dizziness in older children compared with young children and infants were probably caused by increased ability to report symptoms.

This pooled analysis had some limitations. First, as an unplanned pooling of data, the seven studies used various schedules for assessment and procedures for reporting adverse events. Accordingly, the reported rates of events cannot reliably be compared between trials. However, it is unlikely that this limitation impaired detection of any novel safety signals. Second, three of the seven studies were undertaken before 2000, i.e., more than 10 years ago, although there is no evidence to suggest that the efficacy and safety of AL have changed in the past decade^45^ because there are numerous more recent randomized trials confirming its therapeutic effect.^10^,^11^,^14^,^20^,^22^–^25^,^28^,^46^–^48^ Third, five studies were open-label and two were single-arm studies. The open-label designs were used because of the difficulty of using double-blind methods, which would have required large numbers of tablets in these acutely ill patients. Some studies were non-comparative, either because no suitable comparator was available at the time or because inclusion of a control arm would have greatly increased the time required for recruitment. Perhaps less importantly, but still of relevance, supervision of the AL doses was not specified for two trials. Fourth, no adult patients were recruited in Africa, but the studies in children were predominantly in African children. However, there is no evidence in the literature of a difference in PCR-corrected AL cure rates between geographic regions.^60^ Fifth, no children were included in the only study of non-immune persons. Sixth, laboratory data collection was relatively limited and varied between studies, and sampling and analysis were often restricted by local facilities. Sixth, because studies were derived from the Novartis database to permit full access to data, it must be remembered that all trials were supported by Novartis as part of the registration activity for AL in the United States, Europe, Switzerland, and malaria-endemic countries.

In conclusion, in this pooled analysis of almost 2,000 patients, the PCR-corrected 28-day cure rate for AL treatment of uncomplicated *P. falciparum* malaria exceeded 95% in adults and children, and showed a good safety and tolerability profile and rapid reduction in gametocytemia. Although data for non-immune persons remain limited, our findings are consistent with the recommendation that AL be used as first-line treatment of uncomplicated *P. falciparum* malaria in patients of all ages, whether they are semi-immune residents or non-immune visitors in malaria endemic or non-endemic regions.

## Figures and Tables

**Table 1:** Characteristics of studies included in pooled analysis for patients treated for uncomplicated *Plasmodium falciparum* malaria*

Characteristic	Study and reference						
	A025^19^	A026^26^	A028^27^	A2401^36^	A2403^33^	B2303^34^	A2412^35^
Design	Randomized, double-blind, multicenter	Randomized, open-label, multicenter	Randomized, open-label, multicenter	Single-arm, open- label, multicenter	Single-arm, multicenter	Randomized, investigator-blinded, multicenter	Randomized, open-label, single-center
Comparators	4-dose AL	Mefloquine plus artesunate	Mefloquine plus artesunate	–	–	Dispersible AL tablets	Mefloquine plus artesunate, atovaquone plus proguanil
Location	Thailand	Thailand	Thailand	European Union and Colombia†	Africa‡	Africa§	Thailand
Time period	1996–1997	1997–1998	1998–1999	2001–2005	2002–2003	2006–2007	2005
Inclusion criteria							
Age, years	≥ 2	≥ 2	> 12	> 2, ≥ 18 post-amendment	<10	≤ 12	>
Body weight, kg	Not specified	Not specified	≥ 35	Not specified	5–25	5–< 35	Not specified
Fever	Not specified	Not specified	Not specified	Not specified	≥ 37.5°C	≥ 37.5°C axillary or ≥ 38°C rectally or history of fever in previous 24 hours	≥ 37.5°C
Microscopic confirmation of *P. falciparum* infection	Yes	Yes	Yes	Yes	Yes	Yes	Yes
*P. falciparum* density/μL	> 500	> 500	Not specified	Not specified	1,000–< 100,000	2,000–< 200,000	50–< 100,000
AL dosing							
AL dosing	Supervised	Supervised	Not specified	Not specified	Supervised	Supervised	Supervised
Concomitant food/milk	Not specified	Recommended	Recommended	Recommended	Recommended	Recommended	Recommended
Patients included in study populations							
Adult mITT population (n = 599)	180	108	149	162	–	–	–
Adult safety population (n = 647)	180	109	149	165	–	–	44
Children mITT population (n = 877)	59	41	15	–	310	452¶	-
Children safety population (n = 1,332)	59	41	15	–	310	899¶	8

* AL = artemether-lumefantrine; mITT = modified intent-to-treat.

† Non-immune travelers.

‡ Kenya, Nigeria, and Tanzania.

§ Benin, Kenya, Mali, Mozambique, and Tanzania.

¶ Patients receiving the dispersible formulation of AL were only included in the safety population.

**Table 2:** Demographics and baseline disease characteristics of the study populations for patients treated with artemether-lumefantrine for uncomplicated *Plasmodium falciparum* malaria*

Characteristic	Adults		Children	
	Efficacy mITT population (n = 599)	Safety population (n = 647)	Efficacy mITT population (n = 877)	Enrolled population (n = 1,333†)
Male sex, no. (%)	434 (72.5)	472 (73.0)	476 (54.3)	711 (53.3%)
Age (years)				
Mean ± SD	31.2 ± 11.5	31.0 ± 11.4	4.2 ± 4.1	4.1 ± 3.7
Range	17–71‡	17–71§	0–16	0–16
> 0 to ≤ 2, no. (%)	–	–	410 (46.8)	587 (44.0)
> 2 to ≤ 12, no. (%)	–	–	410 (46.8)	680 (51.0)
> 12 to ≤ 16, no. (%)	–	–	57 (6.5)	66 (5.0)
Race, no. (%)				
Caucasian	77 (12.9)	80 (12.4)	0	0
Black	40 (6.7)	40 (6.2)	762 (86.9)	1,209 (90.7)
Asian	0	44 (6.8)	0	9 (0.7)
Other	45 (7.5)	45 (7.0)	0	0
Not collected	437 (73.0)	438 (67.7)	115 (13.1)	115 (8.6)
Body weight (kg)				
Mean ± SD	57.4 ± 13.4¶	57.0 ± 13.2	15.6 ± 9.1	15.3 ± 8.4
Range	34–119¶	34–119#	5.0–58.0	5.0–58.0
5–< 10, no. (%)	–	–	238 (27.1)	323 (24.2)
10–< 15, no. (%)	–	–	309 (35.2)	498 (37.4)
15–< 25, no. (%)	–	–	217 (24.7)	361 (27.1)
25–< 35, no. (%)	–	–	57 (6.5)	87 (6.5)
≥ 35, no. (%)	–	–	56 (6.4)	64 (4.8)
*P. falciparum* parasite count (asexual forms/μL)				
Median	4,618	5,236	23,762	24,178
Range	13–464,880	0–464,880	188–628,571	0–628,571
≤ 100,000, no. (%)	464 (77.5)	505 (78.1)	786 (89.6)	1,183 (88.7)
<2,000, no. (%)	163 (27.2)	176 (27.2)	44 (5.0)	50 (3.8)
2,000–< 5,000	90 (15.0)	92 (14.2)	112 (12.8)	165 (12.4)
5,000–< 15,000	66 (11.0)	92 (14.2)	194 (22.1)	276 (20.7)
15,000–< 50,000	98 (16.4)	110 (17.0)	262 (29.9)	440 (33.0)
50,000–< 100,000	47 (7.8)	51 (7.9)	174 (19.8)	252 (18.9)
> 100,000, no. (%)	34 (5.7)	37 (5.7)	91 (10.4)	147 (11.0)
None, no. (%)	0	4 (0.6)	0	1 (0.1)
Missing/different unit, no. (%)	101 (16.9)	101 (15.6)	0	2 (0.2)
Body temperature (°C)				
Mean ± SD	37.9 ± 1.1	37.8 ± 1.1	38.2 ± 1.0	38.2 ± 1.1
< 37.5, no. (%)	235 (39.2)	267 (41.3)	164 (18.7)	301 (22.6)
37.5–< 39.0, no. (%)	243 (40.6)	255 (39.4)	485 (55.3)	709 (53.2)
≥ 39.0, no. (%)	121 (20.2)	125 (19.3)	227 (25.9)	319 (23.9)
Missing, no. (%)	0	0	1 (0.1)	4 (0.3)
Renal function at baseline**				
Normal, no. (%)	223 (37.2)	225 (34.8)	184 (21.5)	326 (24.5)††
Mild dysfunction, no. (%)	143 (23.9)	143 (22.1)	321 (36.6)	510 (38.3)††
Moderate dysfunction, no. (%)	9 (1.5)	10 (1.5)	191 (21.8)	292 (21.9)††
Severe dysfunction, no. (%)	0 (0)	0 (0)	92 (10.5)	102 (7.7)††
Missing, no. (%)	224 (37.4)	269 (41.6)	89 (10.1)	102 (7.7)††
Hepatic function at baseline‡‡				
Normal, no. (%)	164 (27.4)	167 (25.8)	144 (16.4)	144 (10.8)
Mild dysfunction, no. (%)	121 (20.2)	121 (18.7)	170 (19.4)	170 (12.8)
Moderate dysfunction, no. (%)	60 (10.0)	60 (9.3)	24 (2.7)	24 (1.8)
Severe dysfunction, no. (%)	17 (2.8)	17 (2.6)	2 (0.2)	2 (0.2)
Missing, no. (%)	237 (39.6)	282 (43.6)	537 (61.2)	992 (74.5)

* mITT = modified intent-to-treat.

† One patient in study B2412 did not receive artemether-lumefantrine and was excluded from the safety population.

‡ Three patients were ≥ 65 years of age.

§ Two patients were ≥ 65 years of age.

¶ Study A2401: mean ± SD = 72.9 ± 13.5 kg, range 47–119 kg (66 patients weighed > 70 kg and 5 patients weighed > 100 kg). # One patient weighed < 35 kg.

** Defined by creatinine clearance (Cockcroft-Gault/Shull): normal > 80 mL/minute; mild dysfunction ≥ 50–≤ 80 mL/minute; moderate dysfunction ≥ 30–< 50 mL/minute; or severe dysfunction < 30 mL/minute.

†† Safety population (n = 1,332).

‡‡ Defined as normal*,* either total bilirubin ≤ upper limit of normal [ULN], and aspartate aminotransferase [AST] ≤ ULN or total bilirubin ≤ ULN and AST value missing; mild dysfunction, either total bilirubin ≤ ULN and AST> ULN, or total bilirubin > ULN – 1.5 × ULN regardless of AST value; moderate dysfunction, total bilirubin > 1.5 – 3 × ULN regardless of AST value; severe dysfunction, total bilirubin > 3 × ULN regardless of AST value; missing, baseline for total bilirubin is missing regardless of whether AST value is missing. If baseline AST value was missing, baseline alanine aminotransferase value was used as per the criteria for AST.

**Table 3:** Parasitologic cure rate at day 28 for patients treated with artemether-lumefantrine for uncomplicated *Plasmodium falciparum* malaria*

Group	Adults (all patients)		Adults (Study A2401)		Children	
	No./total (%)	95% CI	No./total (%)	95% CI	No./total (%)	95% CI
mITT population						
Non-PCR corrected	497/599 (83.0)	79.7–85.9	120/162 (74.1)	66.6–80.6	743/863 (86.1)	83.6–88.3
PCR corrected†	499/598 (83.4)	80.2–86.3	120/162 (74.1)‡	66.6–80.6	798/854 (93.4)	91.6–95.0
Evaluable patients						
Non-PCR corrected	494/511‡ (96.7)	94.7–98.1	119/124 (96.0)	90.8–98.7	737/823 (89.6)	87.3–91.6
PCR corrected†	495/510§¶ (97.1)	95.2–98.3	119/124 (96.0)	90.8–98.7	792/814 (97.3)	95.9–98.3

* CI = confidence interval; mITT = modified intent-to-treat; PCR = polymerase chain reaction.

† PCR analysis was not performed in study A2401.

‡ Two patients were excluded from this analysis after starting antimalarial treatments before day 28 (for reasons other than rescue medication).

§ Last observation carried forward: 147 of 162 (90.7%).

¶ One patient was excluded because of a new infection that started before day 28.

**Table 4:** Subpopulation analysis of 28-day PCR-corrected parasitologic cure rate (evaluable population) for patients treated with artemether-lumefantrine for uncomplicated *Plasmodium falciparum* malaria *

Characteristic	Adults (all patients), no./total (%)	Adults (Study A2401), no./total (%)	Children, no./total (%)
Age			
> 1 month–≤ 2 years	–	–	366/377 (97.1)
2 years–≤ 12 years	–	–	374/384 (97.4)
12–≤ 16 years	–	–	52/53 (98.1)
Body weight (kg)			
5–< 10	–	–	210/217 (96.8)
10–< 15	–	–	283/290 (97.6)
15–< 25	–	–	198/204 (97.1)
25–< 35	–	–	50/51 (98.0)
≥ 35	–	–	50/51 (98.0)
Body weight (kg)			
< 70	–	52/52 (100.0)	–
≥ 70	–	66/71 (93.0)	–
Baseline parasite density, no. (%)†			
≤ 100,000/μL	399/408 (97.8)	50/50 (100.0)	718/737 (97.4)
> 100,000/μL	27/28 (96.4)	0/0	74/77 (96.1)
Renal function,‡ no. (%)			
Normal	182/188 (96.8)	87/91 (95.6)	169/173 (97.7)
Mild dysfunction	118/126 (93.7)	27/28 (96.4)	289/302 (95.7)
Moderate dysfunction	9/9 (100.0)	0/0	174/176 (98.9)
Severe dysfunction	0/0	0/0	85/86 (98.8)
Missing	186/187 (99.5)	5/5 (100.0)	75/77 (97.4)
Hepatic function,§ no. (%)			
Normal	146/147 (99.3)	57/58 (98.3)	137/141 (97.2)
Mild dysfunction	101/106 (95.3)	33/35 (94.3)	159/164 (97.0)
Moderate dysfunction	45/48 (93.8)	16/17 (94.1)	21/22 (95.5)
Severe dysfunction	9/13 (69.2)	0/0	1/2 (50.0)
Missing¶	194/196 (99.0)	13/14 (92.9)	474/485 (97.7)

* PCR = polymerase chain reaction. PCR analysis was not performed in study A2401.

† Data on baseline parasite count not available in parasites/microliter for all patients.

‡ According to creatinine clearance (Cockcroft-Gault/Shull): normal, > 80 mL/minute; mild dysfunction, ≥ 50–≤ 80 mL/minute; moderate dysfunction, ≥ 30–< 50 mL/minute; severe dysfunction, < 30 mL/minute.

§ Defined as normal, either total bilirubin ≤ upper limit of normal (ULN), and aspartate aminotransferase (AST) ≤ ULN or total bilirubin ≤ ULN and AST value missing; mild dysfunction, either total bilirubin ≤ ULN and AST > ULN, or total bilirubin > ULN – 1.5 × ULN regardless of AST value; moderate dysfunction, total bilirubin > 1.5 – 3 × ULN regardless of AST value; severe dysfunction, total bilirubin > 3 × ULN regardless of AST value; missing, baseline for total bilirubin is missing regardless of whether AST value is missing. If baseline AST value was missing, baseline alanine aminotransferase value was used as per the criteria for AST.

¶ Total bilirubin value was missing.

**Table 5:** Secondary efficacy endpoints (mITT population) for patients treated with artemether-lumefantrine for uncomplicated *Plasmodium falciparum* malaria*

Endpoint	Adults (n = 599)	Children (n = 877)
Parasite clearance time (hours)		
Median (95% CI)†	42.3 (41.5–43.2)	35.3 (31.7–35.7)
Mean ± SE†	47.3 ± 1.6	33.2 ± 0.6
≤ 48 hours, no. (%)	471 (78.6)	792 (90.3)
> 48 hours, no. (%)	102 (17.0)	60 (6.8)
Not achieved, no. (%)	26 (4.3)	25 (2.9)
Fever clearance time (hours)		
Median (95% CI)†	28.5 (22.3–34.0)	7.9 (7.9–8.0)
Mean ± SE†	37.4 ± 1.8	25.2 ± 1.9
Gametocytes, no. (%)		
Baseline	58/596 (9.7%)	45/877 (5.1%)
> 0–72 hours	90/597 (15.1%)	90/869 (10.4%)
> 72 hours–day 7	42/543 (7.7%)	14/828 (1.7%)
> Day 7	23/554 (4.2%)	8/846 (0.9%)

* mITT = modified intent-to teat; CI = confidence interval. The time at which parasite clearance and fever clearance were assessed varied between studies.

† Kaplan Meier estimates; if the last observation is censored, the mean is underestimated.

**Table 6:** Adverse events in ≥ 5% of adults or children (safety population) treated with artemether-lumefantrine for uncomplicated *Plasmodium falciparum* malaria

Adverse event	Adults (n = 647), no. (%)	Children (n = 1,332), no. (%)
Any	557 (86.1)	970 (72.8)
Headache	360 (55.6)	168 (12.6)
Anorexia	260 (40.2)	175 (13.1)
Dizziness	253 (39.1)	56 (4.2)
Asthenia	243 (37.6)	63 (4.7)
Arthralgia	219 (33.8)	39 (2.9)
Myalgia	206 (31.8)	39 (2.9)
Nausea	169 (26.1)	61 (4.6)
Pyrexia	159 (24.6)	381 (28.6)
Chills	147 (22.7)	72 (5.4)
Sleep disorder	144 (22.3)	27 (2.0)
Palpitations	115 (17.8)	24 (1.8)
Vomiting	113 (17.5)	242 (18.2)
Abdominal pain	112 (17.3)	112 (8.4)
Fatigue	111 (17.2)	46 (3.5)
Hepatomegaly	59 (9.1)	75 (5.6)
Splenomegaly	57 (8.8)	124 (9.3)
Diarrhea	46 (7.1)	100 (7.5)
Cough	37 (5.7)	302 (22.7)
Anemia	23 (3.6)	115 (8.6)
*P. falciparum* infections	13 (2.0)	224 (16.8)

**Table 7:** Adverse events potentially related to hypersensitivity reactions occurring in > 1 adult or > 1 child, according to time after start of artemether-lumefantrine treatment (day 0) (safety population) for uncomplicated *Plasmodium falciparum* malaria

Adverse event	Adults, no. (%)					Children, no. (%)				
Days, preferred term	0–3 (n = 647)	4–10 (n = 625)	11–28 (n = 596)	> 28 (n = 224)	Total (n = 647)	1–3 (n = 1,332)	4–10 (n = 1,306)	11–28 (n = 1,280)	> 28 (n = 910)	Total (n = 1,332)
Any event	18 (2.8)	6 (1.0)	2 (0.3)	1 (0.4)	26 (4.0)	14 (1.1)	13 (1.0)	20 (1.6)	11 (1.2)	56 (4.2)
Rash	16 (2.5)	3 (0.5)	2 (0.3)	1 (0.4)	21 (3.2)	11 (0.8)	9 (0.7)	13 (1.0)	6 (0.7)	38 (2.9)
Urticaria	2 (0.3)	2 (0.3)	0	0	4 (0.6)	1 (0.1)	1 (0.1)	0	0	2 (0.2)
Face edema	0	1 (0.2)	0	0	1 (0.2)	0	1 (0.1)	0	0	1 (0.1)
Dermatitis	0	0	0	0	0	0	0	4 (0.3)	2 (0.2)	5 (0.4)
Eczema	0	0	0	0	0	0	1 (0.1)	1 (0.1)	0	2 (0.2)
Pustular rash	0	0	0	0	0	1 (0.1)	0	1 (0.1)	0	2 (0.2)
Allergic conjunctivitis	0	0	0	0	0	0	0	1 (0.1)	0	1 (0.1)
Atopic dermatitis	0	0	0	0	0	0	0	0	1 (0.1)	1 (0.1)
Infected dermatitis	0	0	0	0	0	0	0	0	1 (0.1)	1 (0.1)
Hypersensitivity	0	0	0	0	0	0	0	1 (0.1)	0	1 (0.1)
Oropharyngeal blistering	0	0	0	0	0	0	0	0	1 (0.1)	1 (0.1)
Maculo-papular rash	0	0	0	0	0	0	1 (0.1)	0	0	1 (0.1)
Face swelling	0	0	0	0	0	1 (0.1)	0	0	0	1 (0.1)
